# Assessing personal recovery in individuals with severe mental illness: validation of the Dutch Brief INSPIRE-O

**DOI:** 10.1007/s00127-025-02815-5

**Published:** 2025-01-31

**Authors:** Wilma E. Swildens, Ellen Visser, Welmoed van Ens, Barbara Schaefer, Annet Nugter, Philippe Delespaul, Jaap van Weeghel, Mike Slade, Sarita A. Sanches

**Affiliations:** 1 Altrecht MHC Institute, Utrecht, The Netherlands; 2 Centre of Expertise in Health and Social Care, Faculty of Health, Sports and Social Work, Inholland University of Applied Sciences, Amsterdam, The Netherlands; 3 Rob Giel Research Center, University Center Psychiatry, University Medical Center Groningen, University of Groningen, Groningen, The Netherlands; 4 Parnassia Group Academy, Parnassia Psychiatric Institute, The Hague, The Netherlands; 5 Mental Health Organization Noord-Holland-Noord, Heerhugowaard, The Netherlands; 6 Mondriaan Mental Health Centre, Heerlen, Maastricht, The Netherlands; 7 Mental Health and Neuroscience (MHeNS), Maastricht University, Maastricht, The Netherlands; 8 Tilburg University, Tilburg, The Netherlands; 9 MIND Netherlands, Amersfoort, The Netherlands; 10 School of Health Sciences, Institute of Mental Health, University of Nottingham, Triumph Road, Nottingham, NG7 2TU UK; 11 Health and Community Participation Division, Faculty of Nursing and Health Sciences, Nord University, Namsos, Norway; 12 Avans University of Applied Sciences, Breda, The Netherlands

**Keywords:** Severe Mental Illness, Patient-rated outcome measures, Research instruments, Personal recovery

## Abstract

**Purpose:**

Recovery is a key objective in mental health services for people with severe mental illness (SMI). In addition to clinical and functional recovery, personal recovery has gained increasing attention. The CHIME Framework identifies five personal recovery processes—Connectedness, Hope, Identity, Meaning, Empowerment—and is the theoretical foundation for the Brief INSPIRE, a validated Patient-Rated Experience Measure (PREM) to evaluate recovery support. Brief INSPIRE was modified to a five-item Patient-Rated Outcome Measure (PROM) assessing recovery, called Brief INSPIRE-Outcome (Brief INSPIRE-O). Subject of this study are the psychometric properties of the Brief INSPIRE-O.

**Methods:**

Data on validity and reliability gathered through annual routine outcome monitoring were collected for 861 individuals with SMI of Flexible Assertive Community Treatment teams and a follow-up measurement was available for 232 of these individuals. Test–retest reliability was evaluated in a separate subset of 30 individuals with SMI.

**Results:**

The Brief INSPIRE-O shows good internal consistency (Cronbach’s alpha 0.77), test–retest reliability, construct validity, sensitivity to change and no floor or ceiling effects. Furthermore, change in Brief INSPIRE-O was positively related to changes in quality of life and negatively to problems in clinical functioning and unmet care need.

**Conclusion:**

Brief INSPIRE-O can be used for research and monitoring to better understand and improve processes of personal recovery in individuals with SMI.

## Introduction

Recovery is a key objective in Mental Health Care (MHC) for people with severe mental illness (SMI), but its definition has changed radically in the last few decades. Originally, recovery was limited to clinical recovery, the reduction of symptoms in individuals with anxiety or no relapse in schizophrenia [1–3]. The concept of clinical recovery does not do justice to the actual experiences of service users. Their recovery experiences lay in daily functioning [4, 5]. This is why, in addition to clinical recovery, functional and personal recovery are included. Functional recovery relates to performance of daily tasks (independent living, work, learning), engaging in social interactions and role performance [6–8]. Personal recovery starts from the perspective of individuals with SMI [9–11]. Reports of personal recovery entail autobiographical, qualitative work. It is the process of regaining control over one’s life and dealing with the challenges of mental illness; an individual journey [12–15]. Or as Anthony [16] puts it: ‘Recovery is a deeply personal, unique process of changing one’s attitudes, values, feelings, goals, skills, and/or roles. It is a way of living a satisfying, hopeful, and contributing life even within the limitations caused by illness’.

Personal recovery, self-assessed, shows only modest to moderate associations with (often professional-rated) functional and clinical recovery [17–19]. Therefore, it is recommended that all recovery domains are included in research [20, 21]. It is important to monitor personal recovery because it reflects the experiences and viewpoints of individuals with SMI and provides hope when clinical recovery is slow [22]. Personal recovery in individuals with SMI is significantly associated with subjective assessment of quality of life [23] as an important Patient-Rated Experience Measure (PREM). Small significant negative associations are also found with the number of unmet needs for care [24].

Several conceptualizations of experiences of personal recovery have been developed [11] of which the CHIME framework by Leamy et al. [10] is widely accepted. This conceptual framework, based on a literature review and narrative synthesis, identified five key recovery processes making the acronym CHIME: (1) Connectedness—having good relationships and being positively connected to others; (2) Hope—the feeling that recovery is possible; (3) Identity—redeveloping a positive sense of self and overcoming stigma; (4) Meaning—living a meaningful life according to one’s own standards and achieving personal goals and fulfilling activities; and (5) Empowerment—having control over one’s life and taking responsibility. This CHIME framework was validated with users of mental health services [25]. Cross-cultural validity was also assessed [26]. In conclusion, the general categories capture key aspects of recovery, applicable across different countries and cultures. The CHIME framework is applied to studies all over the world [18, 21, 27] and offers a theoretical foundation to measures on personal recovery [28].

Shanks et al. [28] and also Felix et al. [29] wrote in their recent reviews that there are many personal self-report measures already, however also state that the psychometric properties of most of these measures are insufficient. Of these existing measures only the 22-item Questionnaire about the Process of Recovery (QPR) [30] is fully consistent with the CHIME framework. According to the review of Shanks et al. [28] the strongest evidence base was for the 50-item Stages of Recovery Instrument (STORI) [31], the 25-item Maryland Assessment of Recovery Scale (MARS) [32], the QPR [30] and the Recovery Assessment Scale (RAS) [33]. The QPR and RAS have, according to Felix et al. [29], the strongest evidence, however none of the measures have high levels of evidence and could be recommended. Another recent study by Vogel et al. [34] compared the RAS, the Dutch 30-item version of the Mental Health Recovery Measure (MHRM) [35] and the 40-item Netherlands Empowerment List (NEL) [36] in relation to the CHIME framework. These authors reached similar conclusions.

To measure recovery using CHIME as basic concept has advantages because of its clear theoretical foundation, which resulted in five key aspects formulated from the point of view of experts by experience. The existing instruments however are quite lengthy and have the disadvantage of inconsistent coverage of the aspects in the CHIME framework. Their application in practice is often challenging, because of the administrative burden for individuals with SMI amongst whom are a large number with mild intellectual disabilities [37].

The INSPIRE [38] is a standardized measure that meets the need for a short, easy to fill in by service users, and reliable instrument that measures personal recovery in clinical practice. Two versions were initially developed: the Full INSPIRE and the Brief INSPIRE. The Full INSPIRE is a 27-item assessment of recovery support, assessing both personally-valued areas of CHIME-related support and therapeutic relationships [38–40]. The Brief INSPIRE is a five-item assessment of recovery support [38, 40] assessing support for each of the five CHIME recovery processes. The Brief INSPIRE does not cover all aspects of the Full INSPIRE but the items were chosen to cover each original CHIME dimension and for their cross-cultural applicability. Both INSPIRE and Brief INSPIRE are Patient-Rated Experience Measures (PREMs), which assess a service user’s perspective on the value of the support they receive from mental healthcare staff. Subjects assess statements such as ‘My worker helps me to feel in control of my life’.

The current study investigated the psychometric properties of the Brief INSPIRE-O, a Patient-Rated Outcome Measure (PROM), adapted from the Brief INSPIRE and intended for use as an outcome measure of personal recovery both for routine use in clinical practice and as a standardized and low-burden outcome measure for research studies. Instead of rating the support of their mental health workers, individuals with SMI are asked to rate their own recovery state, for instance to what extent (not at all–very much) they feel in control of their lives. A Danish version of the Brief INSPIRE-O was recently validated for a group of non-psychotic individuals with SMI [41]. The aim of the current study was to develop and evaluate the reliability, validity and sensitivity to change of the Dutch version of the Brief INSPIRE-O, as a brief measure of personal recovery with individuals with SMI.

## Methods

### Study design

Data were obtained for individuals with SMI at a mental health center located in a predominantly urban area in the middle of the Netherlands between August 2017 and January 2022 as part of the routine outcome procedure to assess progress of service users over time [42, 43]. Recruitment occurred in ten Flexible Assertive Community Treatment Teams (FACT) [44] with a patient population of approximately 3500 persons in this time period. Individuals treated in FACT-teams are individuals with severe SMI defined as having [45]: (1) a SMI diagnosis, (2) enduring limitations in daily functioning related to their psychiatric problems, (3) a need of long term (with a minimum of 2 years) MHC. Routine outcome measurements regarding individuals treated in the participating FACT-teams were conducted annually as well as at the start and at discharge of treatment with a minimum of 6 and a maximum of 18 months between each measurement.

To assess reliability, we assessed the internal consistency and the test–retest reliability of the Brief INSPIRE-O. For construct validity we determined the correlations with other routinely used instruments and tested hypotheses based on studies reported earlier. For convergent validity we hypothesized that the Brief INSPIRE-O would be strongly positively correlated with quality of life measured with the Manchester Short Assessment of Quality of Life (MANSA) [46]. To assess divergent validity, we hypothesized a weaker or no correlation between Brief INSPIRE-O and the number of unmet needs measured with the Camberwell Assessment of Needs (CANSAS) [47] and social disability measured with the Health of the Nation Outcome Scale (HoNOS) [48] and with limitations in clinical disability measured with the HoNOS symptom dimension. To assess sensitivity to change we investigated whether improvement in Brief INSPIRE-O over time is related to improvement in quality of life and decreases in clinical symptoms.

### Measures

The Brief INSPIRE-O self-assesses personal recovery using five items, one item for each of the CHIME recovery processes: Connectedness, Hope, Identity, Meaning and purpose, and Empowerment. It is based on the validated instruments the Full INSPIRE [38, 39] and the Brief-INSPIRE [40] as described in the introduction. The original five items of the Brief INSPIRE (measuring support) were rephrased for the Brief INSPIRE-O to measure recovery outcomes as follows; 1. I feel supported by other people, 2. I have hopes and dreams for the future, 3. I feel good about myself, 4. I do things that are meaningful to me, and 5. I feel in control of my life. Each item is rated on a 5-point Likert scale with 0 = ‘Not at all’, 1 = ‘Not much’, 2 = ‘Somewhat’, 3 = ‘Quite a lot’, and 4 = ‘Very much’. Total scores range from 0 to 20 and are multiplied by five to produce a final score ranging from 0 (no recovery) to 100 (full recovery). The Brief INSPIRE-O was developed in Dutch and back-translated into English according to the B1-Dutch (intermediate) reading level [49]. The instrument’s translation was tested with a panel of experts in SMI including experts by experience. Recently good psychometric properties where reported for a Danish version of the Brief INSPIRE-O [41] tested with non-psychotic individuals in MHC.

The Manchester Short Assessment (MANSA) measures quality of life in individuals with psychiatric problems [46, 50]. The MANSA consists of 12 self-report items that are rated on a 7-point Likert scale ranging from 1 = ‘Very dissatisfied, couldn’t be worse’ to 7 = ‘Very satisfied, couldn’t be better’. The total score ranges from 0 to 84, with higher scores indicating better quality of life. The MANSA showed a satisfactory construct validity and adequate reliability in terms of good internal consistency with a Cronbach’s alpha of 0.70–0.81 in diverse populations.

The Health of the Nation Outcome Scales (HoNOS) was used to measure limitations in clinical and social functioning [48]. The 12-items HoNOS generates a total score for psychosocial functioning. Items are rated by an MHC professional based on clinical conversations with the individual with SMI and scored on a 5-point Likert scale with 0 = ‘No problem’, 1 = ‘Minor problem requiring no action’, 2 = ‘Mild problem but definitely present’, 3 = ‘Moderately severe problem’, and 4 = ‘Severe to very severe problem’. Total scores range from 0–48, with higher scores indicating poorer psychosocial functioning. The Dutch HoNOS showed satisfactory validity [51]. A review on psychometric properties of the HoNOS [52] showed moderate to high internal consistency with alpha of 0.59–0.76. Following examples of earlier studies, symptomatic remission is defined as having no clinically relevant scores (only scores 0 or 1) on the main HoNOS symptom items psychotic symptoms (item 6), depressive symptoms (item 7) or other behavioral/mental problems (item 8) [53].

The Camberwell Assessment of Needs Short Appraisal Schedule (CANSAS) was used to identify service users’ care needs [47]. We used the Dutch extended 25-item version rated by research assistants based on an interview [54]. The CANSAS items are scored each as 0 = ‘no problem or no need for care in this area’, 1 = ‘a met need, no/moderate problem because of ongoing intervention’, 2 = ‘an unmet need, a current serious problem not covered by help’. Summary scores for the CANSAS can be scored as follows: (1) total number of needs; (2) total number of unmet needs; and (3) total number of met needs. The CANSAS Cronbach’s alpha for unmet needs was found to be acceptable with Cronbach alpha of 0.647 [55]. It shows a moderate to high interrater reliability; κ = 0.53 to κ = 0.91 [56]. For extra analyses in this study, we grouped the unmet need scores on a conceptual sub-domain ‘rehabilitation’ with the CANSAS items: day activities, companionship, intimate relationships, sexuality, childcare, gainful employment and meaningful life. We choose to include these specific analyses because the items have overlapping content with Brief INSPIRE-O items.

### Procedures and participants

A multi-informant approach was used. At each measurement occasion, the MHC workers assessed the level of functioning of their service users with the HoNOS. At the same time, the individuals with SMI were invited to participate in an interview by research assistants (certified psychologists) to assess care needs (CANSAS), quality of life (MANSA), and personal recovery (Brief INSPIRE-O). The HoNOS was rated by the central case manager (mental health nurse or social worker).

Of the individuals approached to participate in the interview during our study period 861 completed the Brief INSPIRE-O. Of those individuals who participated in a first measurement (T1 group), 232 also did a follow-up measurement (T2 group). For test–retest analyses of the Brief INSPIRE-O a separate group of 30 individuals with SMI was asked to complete the instrument twice with a two-week interval between measurements. All included individuals with SMI signed informed consent allowing the use of data for research.

### Analysis

All data were analyzed in SPSS-28. Data were described using general descriptive statistics: distribution of items, range, and mean (SD). Mean and total scores were calculated according to the instructions of the measurement instruments. Internal consistency was estimated using Cronbach’s alpha. Test–retest reliability was estimated by computing the correlation between the two assessments. Spearman's correlation coefficients were chosen because of the ordinal and non-normal nature of the data [57]. Differences between respondents with and without a follow-up measurement, and between participants in the main dataset and the test–retest dataset were computed using the Welch T-test and Chi-square tests as the data was not distributed normally [58, 59]. Extra sensitivity analyses were done with Mann–Whitney U and the Fisher's exact tests. For all tests of difference, the statistical significance was set at 5%.

Convergent and divergent validity were assessed by computing Spearman's correlation coefficients between Brief INSPIRE-O and MANSA, HoNOS-total and symptomatic remission scores, and CANSAS scores for unmet needs (total scores and domain rehabilitation scores).

The magnitude of change in Brief INSPIRE-O scores over time (Baseline and follow-up after 6–18 months) was calculated using the Wilcoxon signed-rank test. To measure meaningful change, we further calculated the Effect Size (ES) which can be used to measure change on group level but also on individual level for which an ES of 0.05 is mostly applied [58].

To study the sensitivity to change in personal recovery scores over time, we compared Brief INSPIRE-O scores to the change in other instruments to investigate whether the change over time was in the expected direction. We correlated change in Brief INSPIRE-O with change in clinical functioning as measured with the HoNOS and change in quality of life as measured with the MANSA.

## Results

### Sample and characteristics

In total, Brief INSPIRE-O assessments were completed by 1151 (33%) of the 3493 individuals with SMI that were accessed by their health care workers. 56 individuals were excluded because they did not provide informed consent for research and 234 individuals because they did not fully complete the Brief INSPIRE-O with other instruments. This means that 861 individuals with SMI were included in the study with a first measurement with the INSPIRE-O. These individuals were at the moment of their first INSPIRE-O measurement in MHC for average 8.0 (SD = 7.46) years. Individuals with SMI not participating in an INSPIRE-O interview in the same time period were assessed by their MHC workers as more limited in functioning; a HoNOS score of 9.77 (SD = 6.51; n = 3493) as compared to the score of 8.02 (SD = 5.67; n = 861) in our main study group (t = − 7.297; df = 4734; p < .001). Of this main group of 861 individuals, 232 also completed a follow-up measurement after a period of 6–18 months.

The baseline characteristics of the main study group (n = 861), the follow-up group (n = 232) and of the separate test–retest group (n = 30) are shown in Table 1. The statistics on differences in characteristics between the total sample and the group that participated in the follow-up measurement are presented in Appendix Table 4. Mean age at baseline of the main sample was 44 years (SD = 12.5) and more men (68%) than women (32%) were included. The main psychiatric diagnostic categories were psychosis spectrum disorder (60%), neurodevelopmental disorder/autism (12%) and personality disorder (8%). 73% had a lower or medium level of education, 23% had paid work (those working a few hours included) and 26% volunteer work, 71% lived independently, 29% with supported or sheltered living. Participants in the test–retest were almost twice as likely to have paid work as participants in the baseline group of the main sample (χ^2^(1, N = 865) = 4.894, p = .027) and had higher scores on their first measurement of the Brief INSPIRE-O (T = 2.093; df = 29; p = .045 (Appendix Table 4). Furthermore the group from the main sample with a follow-up measurement had higher scores on baseline quality of life and lower scores on the HoNOS and CANSAS total and unmet needs than the group with only a baseline measurement (Appendix Table 4).

**Table 1:** Sociodemographic and clinical characteristics of individuals with SMI at baseline: main study group (n = 861) and follow-up group  (n = 232) and the separate test–retest group (n = 30)^a^

Variable	Main study group	Follow-up group	Test–retest group
	N = 861	N = 232	N = 30
Mean age (SD)	44.13 (12.51)	44.48 (12.20)	45.33 (11.06)
Sex at birth n (%)			
Male	587 (68.2)	166 (71.6)	19 (63.3)
Female	274 (31.8)	66 (28.4)	11 (36.7)
Main diagnosisn (%)			
Psychotic disorder	514 (59.7)	144 (62.1)	15 (50)
Bipolar disorder	55 (6.4)	10 (4.3)	2 (6.7)
Neurodevelopmental disorders	100 (11.6)	28 (12.1)	5 (16.7)
Depressive or anxiety disorder	59 (6.8)	11 (4.7)	1 (3.3)
Personality disorder	66 (7.7)	21 (9.1)	4 (13.3)
Other^b^	67 (7.8)	18 (7.7)	3 (10)
Educational level			
Low	239 (31.2)	52 (25.1)	11 (36.7)
Medium	320 (41.7)	97 (46.8.)	9 (30.0)
High	207 (27.1)	58 (28.1)	8 (26.7)
Current daytime activities			
Paid employment	195 (22.6)	58 (25)	12 (40)
Unpaid work	220 (25.6)	69 (29.7)	4 (13.3)
Living situation^c^			
Independent	602 (71.3)	172 (76.4)	24 (80)
Dependent	242 (28.7)	53 (23.6)	6 (20)
Brief INSPIRE-O, mean (SD)	58.14 (18.59)	59.46 (16.6)	65.69 (19.12)
HoNOS, mean (SD)	8.02 (5.67)	7.34 (4.88)	7.73 (5.64)
MANSA, mean (SD)	57.18 (12.23)	58.85 (12.32)	60.71 (11.17)
CANSAS, total needs, mean (SD)	7.35 (3.85)	6.91 (3.33)	6.52 (3.43)
CANSAS, unmet needs, mean (SD)	2.56 (2.76)	2.27 (2.60)	2.80 (1.98)
CANSAS, met needs, mean (SD)	4.79 (2.95)	4.64 (2.68)	3.71 (2.51)
CANSAS unmet rehabilitation needs (SD)	0.91 (1.25)	0.83 (1.19)	1.05 (1.15)

^a^Missing values baseline-T1 sample: Educational level n = 95 and Living situation n = 17, HoNOS n = 121, MANSA n = 3, CANSAS = 140. . Missing values follow-up T2-sample Educational level n = 25 , Living situation n = 7, HoNOS n = 17. Missing values test-retest group: Educational level n = 2.

^b^ Category 'other' of the Baseline group consist of the main diagnosis: severe trauma and stress (33); somatization disorder (5), feeding and eating disorder (1), substance addiction disorder (13), dissociative disorder (6), obsessive compulsion disorder (5), unknown/re-assesment (4). Category 'other' of the Follow-up group consists of: severe trauma and stress (6), feeding and eating disorder (1), substance addiction disorder (3), dissociative disorder (1), obsessive compulsion disorder (3), unknown/re-assesment (4). Category 'other' of the Test-retest group consists of: severe trauma and stress (2), substance addiction disorder (1).

^c^Independent living is living alone, or with partner and/or children, with parents and/or family without supported living arrangements. Dependent living is living with supported living arrangements or living in a facility such as a mental health institution, penitentiary facility, facility for homeless people or nursing home.

### Brief INSPIRE-O

On the first assessment, total Brief INSPIRE-O scores of the 861 individuals with SMI ranged from 0 to 100 with an average score of 58.14 (SD = 18.59) (see Table 1); the average item score was 2.33 (SD = 0.74). All response options were used for each item, indicating that Brief INSPIRE-O does not show floor or ceiling effects.

### Internal consistency and test- retest reliability

Internal consistency of the Brief INSPIRE-O was good with a Cronbach’s alpha for the main group at the first measurement (T1) of 0.77 (n = 861). The test–retest reliability measured with the separate test–retest-group (n = 30) after two weeks was also good (r = .88; p < .001).

### Construct validity (convergent and discriminant)

The data on convergent and discriminant validity are presented in Table 2. To study convergent validity, we first correlated the Brief INSPIRE-O with the MANSA.

**Table 2:** Descriptive statistics for the validation measures and their correlation with the Brief INSPIRE-O score (n = 861)

Scale/subscale^a^	Mean (SD)	Range (min–max)	Spearman rho. With BRIEF INSPIRE-O
MANSA	57.18 (12.23)	22.91–84	0.670**
HoNOS total score	8.02 (5.67)	0–30.55	− 0.392**
HoNOS symptomatic remission score	0.38 (0.49)	0–1	0.311**
CANSAS total no or unmet needs	2.56 (2.76)	0–14	− 0.470**
CANSAS no or unmet Rehabilitation needs	0.91 (1.25)	0–6	− 0.405**

^a^For the Brief INSPIRE-O all questions were completed. The other instruments had to be completed for > 80% of the questions. To calculate a total score with < 20% missing or unknown items, the mean on the other items was imputed for the total score on these instruments. The N for the MANSA was 858, for the HoNOS 740 and for the CANSAS 721

**Significant at p < .01 level (two-tailed)

As expected according to our hypotheses in the introduction, Brief INSPIRE-O scores showed a moderate to strong positive relation to the MANSA. To study discriminant validity, we investigated the relationship between the Brief INSPIRE-O and HoNOS. In concordance with our hypotheses, we found a weak to moderate negative relation to HoNOS total scores and weak to moderate positive relation to HoNOS symptomatic remission scores. Finally, discriminant validity was investigated with the scores of the CANSAS focusing on the number of unmet needs and specifically unmet rehabilitation needs. Consistent with our hypotheses, we found a moderate negative relation with the total number of unmet needs and with the number of unmet rehabilitation needs as measured with the CANSAS.

### Sensitivity to change

In the follow-up group of 232 participants with a baseline and a follow-up measurement, the average Brief INSPIRE-O baseline score was 59.46 (SD = 16.61) at T1 and 62.16 (SD = 16.43) at T2 (Z = -2.939, p = .003). The mean time between both measurements was 11.67 months (SD = 2.59). The effect size (ES) showed that in this period 19.4% of participants showed a meaningful decrease in Brief INSPIRE-O scores indicating less recovery, 48.3% of participants did not display a meaningful change, and 32.3% showed a meaningful increase in scores. To investigate sensitivity to change, we calculated if change in the Brief INSPIRE-O correlated with the MANSA and the HONOS (Table 3). As expected, Brief INSPIRE-O scores changed in the same direction as MANSA scores. Improvement on the Brief INSPIRE-O was related to improvement in quality of life with a moderate correlation between change scores. Also, in line with expectations, Brief INSPIRE-O scores changed in the opposite direction to HoNOS scores. An improvement in personal recovery measured with the Brief INSPIRE-O was related to a decrease in limitations in functioning measured with the HoNOS. We did expect these concepts to be only weakly related which was confirmed with the weak correlation between the change scores.

**Table 3:** HoNOS and MANSA change scores and their correlation with Brief INSPIRE-O change scores

Measure	Change from baseline T1 to 6-18 months follow-up T2 Mean (SD)	Range (minimum to maximum)	Correlation with Brief INSPIRE-O change score
MANSA	1.33 (8.58)	− 28 to 36	0.453**
HoNOS	− 0.71 (4.47)	− 15 to 16.18	− 0.285**

**Significant at p < .01 level (two-tailed)

## Discussion

This paper, along with others [41], is set up to evaluate the psychometric properties of the Brief INSPIRE-O which is intended to routinely measure the personal recovery of people with mental health problems. This instrument shows a good internal consistency and a high test–retest reliability within a set of 861 individuals with SMI. Our study further showed good construct validity (convergent as well as discriminant). As expected, higher scores on the Brief INSPIRE-O score were moderately to strongly related to experiencing a higher quality of life measured with the MANSA and Brief INSPIRE-O scores had a small to moderate relation with levels of unmet needs measured with the CANSAS and CANSAS rehabilitation domain. As expected, higher scores on the total Brief INSPIRE-O were moderately related to lower scores on limitations in functioning measured with the HoNOS.

Sensitivity to change was confirmed in a subset of 232 individuals during the 6–18 months follow-up period with an ES for meaningful change of 0.50. A group of 32% displayed meaningful improvement, 48% did not display change and 19% showed a decrease in Brief INSPIRE-O scores. Furthermore looking at the direction of change analyses showed as expected that improvement with the Brief INSPIRE-O was accompanied by improvement in quality of live with the MANSA and a decrease of limitations in functioning measured with the HoNOS.

The good psychometric properties match recent findings of Moeller et al. [41] who validated the Danish version of the INSPIRE-O with a population with the diagnosis major depression, anxiety and personality disorder. Furthermore, although our results regarding the convergent and divergent validity confirm expectations, the interpretation leaves space for speculation. Weak to moderate correlations between personal recovery and clinical recovery have been found in other studies [20]. This could reflect different constructs, but also different perspectives, as clinical recovery in people with SMI is most often assessed by clinicians (e.g. using HoNOS) whereas personal recovery is always measured through self-reports [60]. The limited improvement in personal recovery and other measures can indicate a lack of effective treatment opportunities and/or societal support [61]. A factor can be the fact that we included individuals with SMI with a longer care duration while individuals with a shorter duration tend to improve more in recovery [62]. However, we only used two waves with a period of 6–18 months apart and we have no knowledge yet on longer-term outcomes. De Winter et al. [62] found modest improvement on personal recovery for individuals with SMI in a meta analyses of longitudinal studies with a follow-up of at least 12 months.

## Strengths and limitations

This study has several strengths. First, though several personal recovery instruments exist, we developed the Brief INSPIRE-O because it is most explicitly based on a strong theoretical foundation (CHIME), short enough for routine use, and developed with experts by experience in line with modern psychometric requirements. Second, this short instrument was administered with the support of trained research assistants which gave extra insight in the applicability of the instrument for a vulnerable group of individuals (including some with mild intellectual disabilities). Third, we combined the administration of Brief INSPIRE-O with other instruments including both self-assessment outcomes (quality of life and needs for care) and an outcome measured by the case managers (HoNOS). Fourth, we studied sensitivity to change in a subset of the individuals with SMI over a 6–18-month follow-up period. Finally the study population reflects the characteristics of the SMI group receiving FACT care in the Netherlands [63, 64].

The study also has various limitations. First, we used a sample from an existing practice of routine outcome monitoring in which individuals with SMI can voluntarily decide to participate or not, which has led to the unintentional selection of relatively higher functioning service users according to their HoNOS scores, and a possible bias in results. A second limitation is that the sample for the test–retest calculations was rather small and consisted of patients more often functioning in paid work and already at baseline having better scores on the Brief INSPIRE-O than the total study group.

Fourth, though we worked with a rather large sample size, we believe that further research with larger longitudinal samples of individuals with SMI to investigate long term outcome of Brief INSPIRE-O and its psychosocial predictors is useful.

A final limitation is the fact that besides the MANSA and CANSAS no other specific self-report measure was added to the package of routine outcome monitoring. To investigate convergent validity, it might have been better to also include a self-assessment measure directed at personal recovery such as the QPR.

## Future directions

Our study demonstrates the Brief INSPIRE-O to be a valid short instrument which is suitable for routine outcome monitoring in the Netherlands. The brevity of the Brief INSPIRE-O can be an advantage for routine outcome monitoring when administered to individuals with SMI such as psychotic disorder. The international focus beyond English speaking countries (through multiple translations) and the development of a culturally-informed evidence base of INSPIRE family measures (e.g. through emerging work on global INSPIRE) may be further advantages.

Future studies could choose a more longitudinal time period as personal recovery might need more time. Ideally, information on the care process (psychiatric treatment and recovery-oriented care) could also be included in future studies with the Brief INSPIRE-O. Though such studies are scant and liable to loss to follow-up, they do indicate that at the longer run personal recovery outcomes improve [65]. Research of Thomas et al. [66] among 1739 individuals with SMI showed that personal recovery is promoted by recovery-oriented interventions. Longitudinal predictors of personal recovery to take into account seem to be clinical recovery (symptomatic and functional remission), better baseline quality of life, socio-economic factors such as having an income from work, resistance to stigma, positive self-esteem and less hopelessness [62, 65–68].

Finally, the relevance of the Brief INSPIRE-O for clinical and evaluation purposes requires more attention in future research. Information on individual change on the Brief INSPIRE-O with, for example, the ES statistic should be combined and discussed with information on relevant change from the service user’s perspective. The question if INSPIRE items sufficiently cover the dimensions of the CHIME Framework was critically discussed based on research [39] and the discussion on which items best cover the concept of recovery stays important. Finally since the Brief INSPIRE and therefore also the Brief INSPIRE-O cover only a selection of the Full INSPIRE items, we would recommend to supplement the Brief INSPIRE-O with the Full INSPIRE periodically for an in-depth quality assessment of personal recovery.

## Conclusion

In this study a short new measure of recovery called Brief INSPIRE-O was evaluated, which is intended for routine outcome monitoring and clinical use. The Brief INSPIRE-O is based on a solid theoretical framework (CHIME) and has good psychometric properties. Follow-up studies could focus on longitudinal outcome, to identify the optimal balance between using Brief INSPIRE-O and other research instruments to assess and improve the outcome of recovery-oriented care processes in individuals with SMI and better understand its predictors.

## Appendix

See Table 4.

**Table 4:** Differences at baseline in sociodemographic and clinical characteristics of individuals with SMI of the main study group (n = 861), the follow-up group  (n = 232) and the separate test–retest group (n = 30)

Variable	\Main study group	Follow-up group	Test–retest group	Difference baseline group with and without follow-up	Difference baseline measures of the baseline group and the test–retest group
	N = 861	N = 232	N = 30		
Mean age (SD)	44.13 (12.51)	44.48 (12.20)	45.33 (11.06)	T = 0.506; Df = 424; p = .613	T = 0.584; Df = 31; p = .563
Gender—n (%)					
Male	587 (68.2)	166 (71.6)	19 (63.3)	χ^2^ (1, N = 861) = 1.667, p = .197)	χ^2^ (1, N = 891 = 1.313, p = .576)
Female	274 (31.8)	66 (28.4)	11 (36.7)		
Main diagnosis—n (%)					
Psychotic disorder	514 (59.7)	144 (62.1)	15 (50)	χ^2^ (6, N = 861) = .416, p = 0.255)	χ^2^ (6, N = 891 = 4.058, p = .669)
Bipolar disorder	55 (6.4)	10 (4.3)	2 (6.7)		
Neurodevelopmental disorders	100 (11.6)	28 (12.1)	5 (16.7)		
Depressive or anxiety disorder	59 (6.8)	11 (4.7)	1 (3.3)		
Personality disorder	66 (7.7)	21 (9.1)	4 (13.3)		
Other	67 (7.8)	18 (7.7)	3 (10)		
Educational level					
Low	239 (32.1)	52 (25.1.)	11 (36.7)	χ^2^ (2, N = 766) = 5.220, p = .074)	χ^2^ (2, N = 794) = 1.190, p = .552)
Medium	320 (41.7)	97 (46.8)	9 (30.0)		
High	207 (27.0)	58 (28.0)	8 (26.7)		
Current daytime activities					
Paid employment	195 (22.6)	58 (25.0)	12 (40.0)	χ^2^(1, N = 861) = 1.003, p = .317)	χ^2^ (1, N = 891) = 4.894, p = .027)
Unpaid work	220 (25.6)	69 (29.7)	4 (13.3)	χ^2^ (1, N = 861) = 2.930, p = .087)	χ^2^ (1, N = 891) = 2.300, p = .129)
Living situation					
Independent	602 (71.3)	172 (76.4)	24 (80)	χ^2^ (1, N = 844) = 3.637, p = .057)	χ^2^ (1, N = 874) = 1.702, p = .300)
Dependent	242 (28.7)	53 (23.6)	6 (20)		
INSPIRE, mean (SD)	58.14 (18.59)	59.46 (16.6)	65.69 (19.12)	T = 1.354; Df = 474; p = .176	T = 2.093; Df = 29; p = .045
HoNOS, mean (SD)	8.02 (5.67)	7.34 (4.88)	7.73 (5.64)	T = -2,259; Df = 479; p = .024	T = -254; Df = 25; p = .802
MANSA, mean (SD)	57.18 (12.23)	58.85 (13.23)	60.71 (11.17)	T = 2.571; Df = 452; p = .010	T = 1.696; Df = 31; p = .563
CANSAS, total needs, mean (SD)	7.35 (3.85)	6.91 (3.33)	6.52 (3.43)	T = -2.248; Df = 542; p = .025	T = -1.066; Df = 20; p = .299
CANSAS, unmet needs, mean (SD)	2.56 (2.76)	2.27 (2.60)	2.80 (1.98)	T = -1.898; Df = 489; p = .024	T = -0.544; Df = 21; p = .592
CANSAS, met needs, mean (SD)	4.79 (2.95)	4.64 (2.68)	3.71 (2.51)	T = -0.966; Df = 514; p = .167	T = -1.886; Df = 20; p = .074
CANSAS unmet rehabilitation needs (SD)	0.91 (1.25)	0.83 (1.19)	1.05 (1.15)	T = -1.289; Df = 542; p = .012	T = -0.531; Df = 20; p = .601

Independent Welch T-tests and Chi-squares were performed. Extra sensitivity analyses with Fisher's Exact tests were done on the Chi-square analyses. No differences were found on the significance levels

Extra analyses to supplement the Welch T-tests were done with Mann-Whitney U test. The following differences between the baseline group with and without follow-up are with this extra test no longer significant: HoNOS (U = 69.899; p = .134), CANSAS total needs (U = 52.198; p = .083); CANSAS unmet needs (U = 51.784; p = .055), CANSAS unmet rehabilitation needs (U = 53.475; p = .174)
